# Is There an Association between Health Risk Behaviours and Academic Achievement among University Students?

**DOI:** 10.3390/ijerph18168314

**Published:** 2021-08-05

**Authors:** Catriona Kar Yuen Ong, Melinda J. Hutchesson, Amanda J. Patterson, Megan C. Whatnall

**Affiliations:** 1 School of Health Sciences, College of Health, Medicine and Wellbeing, University of Newcastle, Callaghan, NSW 2308, Australia; catrionakaryuen.ong@uon.edu.au (C.K.Y.O.); Melinda.hutchesson@newcastle.edu.au (M.J.H.); Amanda.patterson@newcastle.edu.au (A.J.P.); 2 Priority Research Centre for Physical Activity and Nutrition, University of Newcastle, Callaghan, NSW 2308, Australia

**Keywords:** university students, college students, lifestyle risk factors, academic achievement

## Abstract

University students have high rates of health risk behaviours, and these may be predictive of academic success. This cross-sectional study aimed to determine the association between individual and multiple health risk behaviours and academic achievement in a sample of Australian university students. Data from the University of Newcastle Student Healthy Lifestyle Survey 2019 were used. Health risk behaviours (diet, physical activity, sitting time, sleep, alcohol consumption, smoking) were assessed, and total number of risk factors calculated. Academic achievement was assessed using self-reported grade point average (GPA). The association between health risk behaviours and GPA was explored using linear regression, adjusted for socio-demographic and student characteristics. The sample included 1543 students (mean age 25.0 ± 7.9 years, 70.6% female). Lower GPA was associated with not meeting fruit consumption recommendations (β = −0.203), consuming >1 cup of soft drink/week (β = −0.307), having takeaway foods ≥1 time/week (β = −0.130), not consuming breakfast daily (β = −0.261), not meeting sleep recommendations (β = −0.163), exceeding single occasion alcohol consumption risk (β = −0.277), smoking (β = −0.393), and having a higher number of risk factors (β = −0.105). This study identified modest associations between GPA and health risk behaviours, suggesting that further research is warranted into whether strategies to improve university students’ health could modestly improve their academic achievement.

## 1. Introduction

The number of students enrolled in university has increased worldwide in the past two decades [1]. In Australia, there were a total of 1.6 million university enrolments in 2018, which has increased by approximately 900,000 students since 2000 [2,3]. Research has shown that students with good academic success or higher levels of university education are more likely to be employed and paid a higher salary, leading to them becoming more successful in their jobs, more confident about their futures, and more active and healthy into their futures [4].

The transitional period from high school to university is a crucial time for students since these individuals need to take on academic and financial responsibilities as well as develop new social networks [5]. Being a phase of life with increased independence and autonomy, this period is critical for developing positive health behaviours (i.e., diet, physical activity, sitting time, sleep, alcohol consumption, smoking) that will impact their health outcomes, including mental health [5,6,7]. Hence, it is vital to examine these health behaviours since they are key modifiable risk factors for many non-communicable diseases [8]. Additionally, these health behaviours may be predictive of academic success, possibly driven by the links between health behaviours and cognitive functioning and memory [9]. For example, physical activity may impact cognitive function through the increased blood flow to the brain during activity stimulating brain angiogenesis and the structures and processes involved in cognitive function [9]. In relation to diet, studies have demonstrated negative cognitive effects of consuming unhealthy diets high in saturated fat and sugar, possibly via processes of neuroinflammation [10]. Drug and alcohol use have been linked with impairments in executive functioning, neuronal damage and decreased angiogenesis [9]. Meanwhile, the link between sleep and cognition is thought to be due to decreased alertness, attention lapses and slowing of cognitive processing while in a sleep deprived state [11].

University students have high rates of poor diet, physical inactivity, long sitting hours, inadequate sleep, excess alcohol consumption, and smoking [12,13]. Unhealthy dietary practices are found to be associated with barriers such as stress, time constraints, high prices of healthy food, and easy access to junk food [7]. For instance, a cross-sectional analysis among 3062 Australian university students has shown that most participants reported lower consumption of nutrient-rich foods and higher consumption of energy-dense nutrient-poor (EDNP) foods compared to dietary guidelines [14]. In the UK, a cross-sectional study conducted across seven universities (*n* = 3706) reported approximately 86% of the sample ate <5 fruit and vegetable servings/day [15].

Physical inactivity is also common among university students and has been found to be associated with factors including older age, lack of social support, and breakfast skipping [16]. A systematic review determining the prevalence of sufficient physical activity among university students across 27 countries (*n* = 35,747) reported that approximately half or more students were insufficiently active in Canada, the United States, China, and Europe [17]. Distinct from physical inactivity is sedentary behaviour, defined as behaviour requiring minimal energy expenditure which is completed while in a sitting, reclining or lying posture e.g., reading while seated [18]. With regards to sedentary behaviour, a systematic review and meta-analysis conducted in 2020 reported estimates across 32 studies, indicating that university students spend an average of 7.29 h/day sitting [19].

Insufficient sleep has also been shown to be a common part of university life and has been linked to mental disorders, risk-taking behaviours, consuming excessive alcohol and caffeine, and high rates of social media use [20]. In cross-sectional surveys examining sleep practices among Australian university students, up to one third of students were found to have insufficient sleep (<6.5 h/night) [20] or reported hours of sleep above or below national recommendations [21].

The high prevalence of alcohol consumption among university students is associated with social influences including persuasion from peers and number of activities or events where alcohol is available [22]. A systematic review of 29 studies examining alcohol consumption among university students in Ireland and the UK has shown almost two-thirds of the sample reported a hazardous alcohol consumption based on the Alcohol Use Disorders Identification Test (AUDIT) [23]. In Australia, a web-based survey of 7237 university students reported 34% met the AUDIT criteria for hazardous alcohol consumption [24]. Smoking among university students is found to be predicted by a lack of coping resources, poor health attitude, and lack of knowledge of the effect of smoking on health [25]. The prevalence of smoking is decreasing in industrialized countries including Australia, for example a cross-sectional survey among Australian university students (*n* = 3077) reported that 7% of the sample identified as current smokers [21].

There is growing evidence demonstrating associations between health risk behaviours (i.e., diet, physical activity, sitting time, sleep, alcohol consumption, smoking) and academic achievement. For example, a recent systematic review identified five cross-sectional and cohort studies that demonstrated positive associations between diet, namely breakfast consumption, eating regular meals, and meeting fruit intake recommendations with students’ academic achievement [26]. Evidence for the relationships between physical activity and sedentary behaviour with academic achievement is inconsistent. For example, a cross-sectional study from Saudi Arabia showed a significant positive association between college students’ physical activity habits and higher GPA [27], whereas a study among 120 undergraduates in Spain found no relationship between physical activity, total sedentary time, total standing time, or total number of sedentary breaks and academic achievement [28]. With regards to sleep, an American study of approximately 3800 college students followed up over five years found that those experiencing chronic sleep deprivation during one or more years of college were up to 40% less likely to graduate within four years [29]. A two-year cohort study in Spain found university students who were heavy episodic drinkers had two-times higher odds of low academic achievement (mean grade below 20th percentile) at the two year follow up [30]. Additionally, a Norwegian cross-sectional study reported high academic achievement to be inversely associated with smoking and snuffing among university students [31].

While there is evidence to support the association of health behaviours with academic achievement, some studies are of low methodological quality, e.g., due to non-validated and subjective assessment of health risk behaviours or academic outcomes [26,27], while others include small sample sizes [28]. There is also a much larger and stronger body of evidence for some health behaviours (e.g., alcohol, sleep) than others (e.g., diet, sedentary behaviour). Furthermore, while studies have looked at individual health risk behaviours and their associations with academic achievement, few have considered more than one or two health behaviours in their analyses. It is important to acknowledge that most university students are likely to demonstrate more than one health risk behaviour since evidence shows that these health behaviours (i.e., diet, physical activity, sitting time, sleep, alcohol consumption, smoking) interrelate [32].

Therefore, the present cross-sectional analysis aims to determine the association between individual health risk behaviours (diet, physical activity, sitting time, sleep, alcohol consumption, smoking), and the sum total of these health risk behaviours, with academic achievement (GPA) among university students in Australia.

## 2. Materials and Methods

### 2.1. Study Design

This study was a secondary analysis of an online cross-sectional survey, the 2019 University of Newcastle (UON) Student Healthy Lifestyle Survey (SHLS). The primary aim of the SHLS was to identify students’ lifestyle-related health risk factors including diet, physical activity, sitting time, sleep, alcohol consumption, and smoking [21]. The conduct and reporting of this study are consistent with the Strengthening the Reporting of Observational Studies in Epidemiology (STROBE) guidelines [33].

### 2.2. Participants and Recruitment

All students of the UON were eligible to participate in the survey. The UON is a large urban university with six campuses, including the main campus in Newcastle, New South Wales (NSW), Australia, four smaller campuses across NSW, and one campus in Singapore. Students were invited to complete the survey through several recruitment mediums: (i) Students enrolled at the UON as of the 9 September 2019 (*n* = 34,924) were sent an email invitation to participate in the survey with two reminder/thank you emails one and two weeks after the original email, (ii) students were made aware of the survey via advertisements posted on the university’s student communication channels, including student social media accounts, screen savers on all university computers, digital signage on campuses, and posters on noticeboards of two campuses, and (iii) lecturers and course coordinators were requested to advertise the survey in class and/or on the online learning management system. The survey was open from 9 September 2019 to 5 October 2019. Upon completion of the survey, participants could enter a prize draw to win one of five gift vouchers valued at AUD 100. This study was approved by the UON Human Research Ethics Committee (H-2015-0459).

### 2.3. Measures

#### 2.3.1. Exposures

##### Dietary Intake

Dietary intake was assessed using short diet questions from the NSW Adult Population Health Survey [34,35]. These questions included “How many serves of fruit/vegetables do you usually eat each day?”, “How many cups of soft drink/cordial/sport drink do you usually drink?”, “How often do you have meals or snacks such as burgers, pizza, chicken or chips from places like McDonalds, Hungry Jacks, Pizza Hut etc.?”, “How often do you usually have something for breakfast?”. These questions have good relative validity and consistency compared with other dietary assessment tools [35,36,37]. Students’ dietary intake was compared to the recommendations of the Australian Dietary Guidelines (ADG) [38].

##### Physical Activity

Duration (minutes) and frequency of participation in different types of activities including walking, moderate, and vigorous exercise in a week were assessed using the Active Australia Survey [39]. The Active Australia Survey has been used nationally and demonstrates good reliability, validity, and acceptability [39]. Sufficient physical activity level was defined as per Australia’s Physical Activity and Sedentary Behaviour guidelines for adults (i.e., ≥150 min/week) [40].

##### Sitting Time

Sitting time was assessed using questions from the NSW Adult Population Health Survey [35]. These questions determine the average time spent sitting on a weekday and a weekend day. The average of total sitting time was calculated as ([total sitting time on a weekday × 5] + [total sitting time on a weekend day × 2])/7 and reported in hours/day. Participants were categorised as sitting <8 h/day and >8 h/day, based on the evidence of greater mortality risk for higher sitting time in comparison with <8 h/day [41].

##### Sleep

Sleep was evaluated using one question developed by the National Centre for Chronic Disease Prevention and Health Promotion [42]; average number of hours of sleep in a 24 h period. Students were considered to be meeting sleep recommendations if: aged 17 years and sleeping 8 to 10 h/day, 18 to 64 years and sleeping 7 to 9 h/day or aged ≥65 years and sleeping 7 to 8 h/day according to the Sleep Health Foundation recommendations [43].

##### Alcohol

Intake of alcohol was assessed as frequency and quantity of consumption, i.e., the number of standard drinks consumed per drinking occasion [44]. Participants who reported consuming ≥4 standard drinks per single occasion were categorised as “exceeding single occasion risk” for alcohol consumption [45].

##### Smoking

Current smoking status was assessed using a question from the NSW Adult Population Health Survey (“which of the following best describes your smoking status?”) [35]. Students who identified themselves as smokers (“I smoke daily” and “I smoke occasionally”) were categorised under “smoker”, while students who identified as ex-smokers (“I don’t smoke now, but I used to”) and non-smokers (“I’ve never smoked”) were categorised under “non-smoker”.

##### Multiple Health Behaviour Risk Factor Score (Risk Factor Score)

In defining a Risk Factor Score, all six health behaviours (i.e., diet, physical activity, sitting time, sleep, alcohol consumption, smoking) were included. Participants were given half to one point if they did not comply with population-based recommendations (occurrence of risk factor) and zero points if they did (no occurrence of risk factor), which were then added to provide a total Risk Factor Score ranging from zero to seven. Consequently, participants were given one point for: (i) not meeting core foods consumption recommendations (0.5 points for not meeting the ADG fruit consumption recommendations, 0.5 points for not meeting the ADG vegetable consumption recommendations); (ii) exceeding discretionary foods consumption recommendations (0.5 points for consuming >1 cup of soft drink per week, 0.5 points for consuming takeaway foods ≥ 1 time per week); (iii) not meeting Australia’s Physical Activity and Sedentary Behaviour guidelines; (iv) having an average sitting time of >8 h/day; (v) not meeting sleep recommendations for age; (vi) exceeding single occasion alcohol consumption risk; (vii) smoking. Lower Risk Factor Score indicates greater compliance with population-based recommendations.

#### 2.3.2. Outcome: Academic Achievement

Academic achievement was assessed as grade point average (GPA) which was self-reported by students on a scale from 0 to 7, to one decimal place. GPA was used as a measure of academic performance as it has been used in research internationally, and self-reported GPA has previously been found to be valid [46,47]. High academic achievement is indicated by a GPA of 7, which is equivalent to a high distinction average.

#### 2.3.3. Socio-Demographics and Student Characteristics

Socio-demographic data collected included students’ age, gender, country of origin, Aboriginal or Torres Strait Islander (ATSI) descent, domestic/international enrolment, living situation, hours of paid work per week, and financial support. Key student-related data collected included student type, faculty of enrolment, and number of years studying.

### 2.4. Statistical Analysis

**Figure 1:**
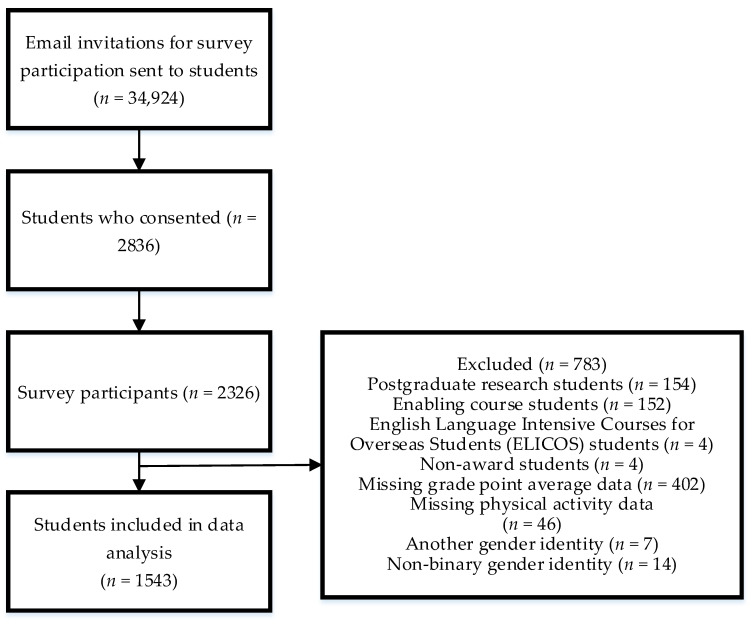
Participant exclusion flowchart for statistical analysis.

Statistical analysis was performed using SPSS statistical software (IBM Corp., Version 24.0. Armonk, USA). Exposure measures (except the Risk Factor Score), socio-demographics, and student characteristics were described as numbers and percentages for categorical variables and as the mean and standard deviation (SD) for continuous variables. GPA and Risk Factor Score were described as the median and inter-quartile range (IQR) due to non-normal distribution. In total, 2836 participants consented to participate in the survey, of which 2326 students were eligible. A total of 1543 students were included in this analysis (see Figure 1). Excluded participants were postgraduate (research higher degree) students (*n* = 154) as they do not receive grades, enabling (i.e., transition to university) course students (*n* = 152), English Language Intensive Courses for Overseas Students (ELICOS) students (*n* = 4), and non-award students (*n* = 4) as they are not graded by GPA, and students with missing data for GPA (*n* = 402) or physical activity (*n* = 46). Participants who reported as “another gender identity” (*n* = 7) and “non-binary” (*n* = 14) were not included due to the small number of participants within these groups. The 402 participants with missing GPA data were not significantly different in their socio-demographic or student characteristics to the included sample. The potential confounders were selected based on several factors including our prior research in this group [14,48] and review of the background literature to identify known determinants of health behaviours and academic achievement. To identify confounders, GPA and health risk behaviours were regressed onto each socio-demographic variable and student characteristic using simple linear regression for GPA and binomial logistic regressions for health risk behaviours. Any socio-demographic or student characteristic that was statistically significant at *p* < 0.2 were included within the final regression model. Multiple linear regression analyses were then used to explore the association between individual health behaviours (i.e., dietary intake, physical activity, sitting time, sleep, alcohol consumption, smoking), the Risk Factor Score, and academic achievement (GPA), with statistical significance set at *p* < 0.05. In the final model, some socio-demographic and student characteristics were excluded by SPSS automatically due to multi-collinearity (see Supplementary Table S1).

## 3. Results

### 3.1. Summary of Sample Characteristics

Students’ socio-demographic characteristics are summarised in Table 1. The mean (SD) age of participants was 25.0 (7.9) years. The majority of participants were female (70.6%), Australian-born (86.6%), undergraduate students (87.9%). Four percent of the sample were of Aboriginal and Torres Strait Islander (ATSI) descent. Students were from all five faculties across the university, mostly enrolled in the Faculty of Health and Medicine (29.3%), and in their first year of study (36.4%). The majority of the participants were living in their parents’ home (39.1%) or rented accommodation (36.2%). The median (IQR) GPA was 5.7 (5.0–6.2).

Table 2 summarises data for the six health risk behaviours. Students who were not meeting the fruit and vegetable consumption recommendations were 52.0% and 90.2%, respectively. Twenty-five percent reported consuming >1 cup of soft drink(s) per week and thirty-five percent reported having takeaway foods ≥1 time(s) per week. Only half of the participants (51.9%) reported having breakfast on a daily basis. Close to one-quarter of the participants were not meeting physical activity recommendations (27.5%) and sleep recommendations (22.6%). Over a third of participants were sitting >8 h/day (35.5%) and exceeding single occasion alcohol consumption risk (33.1%). Seven percent of the participants identified themselves as smokers. The median (IQR) Risk Factor Score (out of a maximum score of seven) was 2.0 (1.0–2.5).

### 3.2. Associations of Academic Achievement (GPA) with Individual Health Risk Behaviours

Key results of the unadjusted and adjusted linear regression models are presented in Table 3, with the full results provided in Supplementary Material Table S1. In the unadjusted models, lower GPA was associated with not meeting fruit recommendations (β = −0.221, *p* ≤ 0.001), not meeting vegetable recommendations (β = −0.201, *p* = 0.037), consuming >1 cup of soft drink per week (β = −0.355, *p* ≤ 0.001), having takeaway foods ≥ 1 time per week (β = −0.169, *p* = 0.005), and not consuming breakfast on a daily basis (β = −0.303, *p* ≤ 0.001). Lower GPA was also associated with not meeting age-based sleep recommendations (β = −0.138, *p* = 0.043), exceeding single occasion alcohol consumption risk (β = −0.299, *p* ≤ 0.001), and smoking (β = −0.417, *p* ≤ 0.001).

In the adjusted models controlling for various socio-demographic and student characteristics (see Supplementary Material Table S1), lower GPA remained significantly associated with not meeting fruit recommendations (β = −0.203, *p* ≤ 0.001), consuming > 1 cup of soft drink per week (β = −0.307, *p* ≤ 0.001), having takeaway foods ≥ 1 time per week (β = −0.130, *p* = 0.029), not consuming breakfast on a daily basis (β = −0.261, *p* ≤ 0.001), not meeting age-based sleep recommendations (β = −0.163, *p* = 0.018), exceeding single occasion alcohol consumption risk (β = −0.277, *p* ≤ 0.001), and smoking (β = −0.393, *p* = 0.001). Applying Bonferroni correction (i.e., 0.05/number of tests) to account for multiple comparisons would give a corrected *p*-value of 0.005 for significance. Using the corrected *p*-value, the associations of sleep and takeaway foods consumption with GPA would no longer be significant.

### 3.3. Associations of Academic Achievement (GPA) with Multiple Health Risk Behaviours (Risk Factor Score)

In the unadjusted models, having a higher number of risk factors (higher Risk Factor Score) was found to be associated with older age, being male, ATSI descent, international enrolment, enrolment in the faculty of Business and Law, Education and Arts, and Engineering and Built Environment, being in the second year of program of study, and not receiving financial support (see Supplementary Material Table S1). In the unadjusted models, lower GPA was associated with having a higher Risk Factor Score (β = −0.111, *p* ≤ 0.001). This association remained significant in the adjusted model controlling for the socio-demographic and student characteristics mentioned above (β = −0.105, *p* ≤ 0.001).

## 4. Discussion

This study aimed to examine the association between health risk behaviours (poor diet, physical inactivity, long sitting time, inadequate sleep, higher risk alcohol consumption, smoking) and academic achievement in a sample of Australian university students. Results showed significant associations between lower GPA and not meeting fruit recommendations, consuming >1 cup of soft drink per week, consuming takeaway foods ≥1 time(s) per week, not eating breakfast on a daily basis, not meeting age-based sleep recommendations, exceeding single occasion alcohol consumption risk, smoking, and having a higher number of health risk behaviours.

Not meeting the ADG fruit consumption recommendations was associated with lower GPA (β = −0.203). This finding is in accordance with Peltzer et al. cross-sectional study of 17,789 university students from 26 countries, which found higher academic achievement in students who met fruit recommendations [49]. In the present study, lower GPA was also found in participants not consuming recommended serves of vegetables; however, this association was only significant in the unadjusted analysis. This finding is in contrast with a study conducted among 16,000 US students that found a 0.15 higher cumulative grade in students consuming ≥ 5 versus < 5 fruit and vegetable serves per day [50]. Variation in definitions of a vegetable serve and consumption recommendations between countries could explain the difference in findings. Lower GPA was also shown in students consuming >1 cup of soft drink per week and takeaway foods ≥1 time(s) per week. This is in line with several existing studies that looked at school-aged children, adolescents, and university students [31,51,52,53]. For instance, a Canadian study (*n* = 5200) reported that children whose diets were characterised by greater intakes of sugar-sweetened beverages (SSBs) and fast food/takeaway meals had poorer academic outcomes [53]. This finding is also consistent with an online survey conducted among Australian university students (*n* = 278) that reported an association between lower GPA and higher percentage energy per day from EDNP foods and SSBs [48]. Further, the present study found that not eating breakfast on a daily basis was significantly associated with lower GPA (β = −0.261). This is in line with studies conducted among adolescents and college students, including longitudinal studies, showing that more frequent breakfast consumption is positively associated with academic achievement [26,54,55].

The present study also demonstrated an association between not meeting sleep recommendations and lower GPA. This is in agreement with studies conducted among college students in other countries, including longitudinal studies [54,56,57]. For example, longitudinal studies among Swiss and Hong Kong university students with one to 15 months of follow up found associations between better sleep quality and longer sleep duration on study days with better academic achievement, while sleep disturbances was associated with poorer academic achievement [54,56,57]. This highlights the importance of adequate sleep in overall concentration and academic achievement, since sleep is known to have an impact on learning, memory, and cognitive function [50]. Overall, the evidence demonstrates that it is not only sleep duration that is an important indicator of sleep health, but the range of factors such as sleep quality, which will impact students’ academic performance [55]. Therefore, it is notable that the present study looked at total sleep hours and not sleep quality or consistency as a predictor variable.

Exceeding single occasion alcohol consumption risk (> 4 standard drinks per drinking occasion) was associated with lower GPA. This result ties well with previous studies wherein students who consumed alcohol at hazardous levels were more likely to experience academic problems which posed a negative impact on learning [58,59]. This is also in agreement with a study that found strong associations between heavy episodic drinking and subjective academic performance i.e., the more often students had ≥5 drinks per drinking occasion, the lower they rated their academic performance [59]. Some studies that have used self-reported GPA as an outcome measure have found significant negative associations between heavy alcohol use and GPA, [60] while in other studies, when multiple factors were controlled, this association was no longer significant [61,62]. However, direct comparisons of findings of the present study with other studies are difficult due to the diversity of approaches used in measuring alcohol use and academic achievement and differences in standard drinks guidelines in various countries.

The present study also found that being a current smoker was associated with lower GPA. To our knowledge, very few studies have explored the association between smoking and academic achievement explicitly. Nevertheless, this finding is consistent with existing studies that reported a relationship between poor academic performance and substance use, including cigarette smoking [63,64,65]. In particular, one study conducted among 477 Spanish university students found the combined use of cannabis and tobacco to be moderately related to poor academic achievement [63]. Existing studies suggest a complex rather than straightforward relationship between smoking and academic achievement that interacts with, or is mediated by students’ psychosocial context, [66,67] presenting opportunities for deeper research into the interrelationship and determinants of smoking and academic achievement.

The present study is one of the first to explore the relationship between multiple health risk behaviours and academic achievement. Lower GPA was found among students with a higher Risk Factor Score, such that GPA was 0.1 lower for the occurrence of every extra health risk behaviour. These findings suggest that the more health risk behaviours a student has, the lower his/her GPA. In other words, non-compliance with public health recommendations was found to be associated with lower GPA, after controlling for various socio-demographic and student-related characteristics.

Interestingly, meeting national physical activity recommendations and having an average sitting time of more than 8 h/day was not significantly associated with GPA. On the contrary, a cross-sectional study among 409 medical students found a significant positive association between students’ physical activity habits and high GPA [27]. It was explained that regular participation in physical activity is linked with enhancement of brain function, thus resulting in a positive impact on academic achievement [27,68]. However, there are also studies that showed consistent findings with the present study, reporting no association between total sedentary time and academic achievement among college students and children [28,69]. It could be hypothesised that when students spend more time exercising, they have less time for their academic work, which might have offset the benefits of physical activity on academic performance [70]. Additionally, it is likely that students who spend more time studying at their desks have longer sitting time.

It is important to acknowledge that given that the results demonstrate small associations, i.e., small differences in GPA, in association with each health risk behaviour and the total Risk Factor Score, the findings of this study may not be particularly meaningful. Values were in the range of 0.01–0.393 lower GPA in adjusted models in students reporting the health risk behaviour versus not, which is small relative to the range of the GPA measure (0–7). It could also be that the behaviours found to be associated with higher GPA are merely an indicator shared by students who are likely to perform better academically. However, it could also be hypothesised that within a larger and more representative sample, larger effects may be observed. This warrants further investigation in studies that observe changes in these health risk behaviours over time while taking into consideration all aspects of the health behaviours.

The main strengths of the present study include the use of validated assessment tools for health risk behaviours and the consideration of a wide set of socio-demographic and student-related characteristics to be adjusted as potential confounders in the statistical analyses. Additionally, self-reported GPA has been found to be a valid measure of academic achievement [47]. However, it should also be considered that the sample was a convenience sample and the GPA of the sample was skewed towards higher values, i.e., indicating that the sample included more high achieving students and may not be representative of the whole student body. In terms of limitations, the SHLS is a self-report survey; hence, the accuracy and reliability of the findings should be interpreted carefully. However, factors such as the participants being anonymous and the use of validated tools may have helped to limit the potential bias from self-reporting. Next, the present study only considered indicators for each health risk behaviour and did not comprehensively assess them. Another important limitation is the Risk Factor Score scoring system that puts more emphasis on dietary intake than other health risk behaviours. Findings related to the Risk Factor Score should be interpreted carefully since all health risk behaviours are not equal. While a range of potential confounders were considered, there are more that could be significant in the association between health risk behaviours and academic achievement, such as indicators of personality (e.g., conscientiousness and neuroticism) and alternative indicators of socio-economic status (e.g., income). Regarding socio-economic status as a confounder, this study used a measure of whether or not students received financial support as an indicator to their socio-economic status. A more direct measure may have been more appropriate; however, the challenge in accurately or directly assessing socio-economic status in this population group should be acknowledged, e.g., due to transient living arrangements, employment and income. Finally, due to the nature of the cross-sectional study design, no causal conclusions can be drawn from the findings nor any extrapolations to longer-term academic achievement or related career success.

The present study has demonstrated small associations between various health risk behaviours and self-reported academic achievement. This can help to improve our understanding of health-promoting behaviours and the intellectual wellbeing of university students. In terms of future observational studies, the findings of the present study support that more in-depth research should be undertaken to provide more information on the associations between changes over time in health risk behaviours and academic achievement, and which aspects of the health risk behaviours may be associated with academic achievement.

## 5. Conclusions

In conclusion, a small yet non-negligible association was found between academic achievement and individual health risk behaviours (i.e., poor diet, inadequate sleep, alcohol consumption, smoking) as well as having a high number of health risk behaviours. These findings support the need for further research to explore these associations more deeply, including changes over time.

## Figures and Tables

**Table 1:** Socio-demographic and student characteristics in a sample of Australian university students (*n* = 1543).

	Mean ± SD or % (*n*)
**Age (years), mean ± SD**	25.0 ± 7.9
**Gender, % (*n*)**	
Female	70.6 (1090)
Male	29.4 (453)
**Country of origin, % (*n*)**	
Australia	86.6 (1337)
Other countries	13.4 (206)
**Aboriginal or Torres Strait Islander, % (*n*)** ^a^	4.1 (64)
**Domestic / International, % (*n*)**	
Domestic	94.2 (1453)
International	5.8 (90)
**Student type, % (*n*)**	
Undergraduate	87.9 (1356)
Postgraduate (coursework)	12.1 (187)
**Faculty of enrolment, % (*n*)**	
Business and Law	15.1 (233)
Education and Arts	25.6 (395)
Engineering and Built Environment	12.9 (199)
Health and Medicine	29.3 (452)
Science	17.1 (264)
**Number of years studying, % (*n*)**	
1st year	36.4 (561)
2nd year	21.3 (328)
3rd year	21.2 (327)
4th year	12.2 (189)
5th year and above	8.9 (138)
**Living situation, % (*n*)**	
Own home	13.0 (201)
Parents’ home	39.1 (604)
On-campus residences	9.0 (139)
Renting	36.2 (558)
Others	2.7 (41)
**Paid work (hours/week), mean ± SD**	13.9 ± 12.5
**Receiving financial support, % (*n*)**	42.0 (648)
**Grade Point Average, median (IQR)**	5.7 (5.0–6.2)

^a^*n* = 1538 (*n* = 5 did not provide information on ATSI descent). SD; standard deviation, IQR; inter-quartile range.

**Table 2:** Health risk behaviours in a sample of Australian university students (*n* = 1543).

	% (*n*) or Median (IQR)
**Dietary intake**	
**Fruit and vegetable consumption**	
Not meeting fruit recommendations	52.0 (802)
Not meeting vegetable recommendations	90.2 (1392)
**Soft drink (cups/day or week)**	
≤1 per week	74.4 (1148)
2–6 per week	20.3 (314)
1 per day	2.5 (38)
≥2 per day	2.8 (43)
**Takeaway foods (times/week)**	
<1	64.6 (997)
1–2	28.6 (442)
3–4	5.9 (91)
5–6	0.7 (11)
Daily	0.1 (2)
**Breakfast consumption (times/week)**	
<1	14.7 (227)
1–2	9.0 (139)
3–4	10.8 (167)
5–6	13.5 (209)
Daily	51.9 (801)
**Physical activity**	
Not meeting physical activity recommendations	27.5 (425)
**Sitting time**	
Sitting >8 h/day	35.5 (547)
**Sleep**	
Not meeting age-based sleep recommendations	22.6 (348)
**Alcohol**	
Exceeding single occasion alcohol consumption risk	33.1 (510)
**Smoking**	
Smoker	6.8 (105)
**Risk Factor Score**	2.0, 1.0–2.5

**Table 3:** Linear regression results of academic achievement (GPA) with health risk behaviours, adjusted for socio-demographic and student characteristics in a sample of Australian university students (*n* = 1543).

Variable	Model	β-Coefficient ^a^	SE	R^2^	*p*
Not meeting fruit recommendations	Unadjusted	−0.221	0.057	0.010	**<0.001**
	Adjusted ^b^	−0.203	0.057	0.047	**<0.001**
Not meeting vegetable recommendations	Unadjusted	−0.201	0.096	0.003	**0.037**
	Adjusted ^c^	−0.125	0.097	0.033	0.197
Soft drink > 1 cup(s) per week	Unadjusted	−0.355	0.065	0.004	**<0.001**
	Adjusted ^d^	−0.307	0.066	0.048	**<0.001**
Takeaway food ≥ 1 time(s) per week	Unadjusted	−0.169	0.060	0.005	**0.005**
	Adjusted ^e^	−0.130	0.060	0.044	**0.029**
Breakfast consumption < daily	Unadjusted	−0.303	0.057	0.018	**<0.001**
	Adjusted ^f^	−0.261	0.057	0.054	**<0.001**
Not meeting physical activity recommendations	Unadjusted	−0.039	0.064	0.0002	0.543
	Adjusted ^g^	−0.010	0.064	0.041	0.877
Sitting time >8 h/day	Unadjusted	−0.030	0.060	0.0002	0.612
	Adjusted ^h^	−0.011	0.060	0.029	0.851
Not meeting age-based sleep recommendations	Unadjusted	−0.138	0.068	0.003	**0.043**
	Adjusted ^k^	−0.163	0.068	0.028	**0.018**
Exceeding single occasion alcohol consumption risk	Unadjusted	−0.299	0.060	0.016	**<0.001**
	Adjusted ^i^	−0.277	0.062	0.054	**<0.001**
Smoker	Unadjusted	−0.417	0.113	0.009	**<0.001**
	Adjusted ^j^	−0.393	0.113	0.033	**0.001**
Risk Factor Score	Unadjusted	−0.111	0.025	0.019	**<0.001**
	Adjusted ^l^	−0.105	0.026	0.048	**<0.001**

^a^ β-Coefficient indicates the decrease in grade point average per unit increase in the independent variable. All adjusted models adjusted for age and gender. Adjusted for international enrolment ^b, c, d, e, f, g, i, l^. Adjusted for student type ^b, d, e, f, g, i^. Adjusted for faculty of enrolment ^b, c, d, e, f, g, h, i, l^. Adjusted for number of years studying ^d, f, g, h, i, j, k, l^. Adjusted for living situation ^b, c, d, e, f, g, h, i, j, k^. Adjusted for paid work ^d, h^. Adjusted for financial support ^b, c, g, l^. Significant *p*-values are indicated in bold. SE—standard error, R^2^—R-squared.

## Supplementary Materials

The following are available online at https://www.mdpi.com/article/10.3390/ijerph18168314/s1. Table S1: Linear regression results of academic achievement (GPA) with health risk behaviours, adjusted for socio-demographics and student characteristics in a sample of Australian university students (*n* = 1543).
