# Supplementary material for: Is There an Association between Health Risk Behaviours and Academic Achievement among University Students?
Source: Int J Environ Res Public Health. 2021 Aug 5;18(16):8314. doi: 10.3390/ijerph18168314 (PMC8392864; doi:10.3390/ijerph18168314)
Supplement: Supplementary file 1 [file ijerph-18-08314-s001.zip › ijerph-1319052-supplementary.pdf]

Table S1. Linear regression results of academic achievement (GPA) with heath risk behaviours, adjusted for socio-demographics and student characteristics in a sample of Australian university students (n = 1543).

Modifiable Lifestyle Risk Factor	Academic Achievement (GPA)		
	**β-Coefficient** [a]	**SE**	**p**
Not meeting fruit recommendations	-0.203	0.057	**< 0.001**
Age	0.008	0.005	0.097
Male	-0.215	0.066	**0.001**
International	-0.323	0.129	**0.013**
Postgraduate	0.333	0.096	**0.001**
Faculty of study			
Reference: Health & Medicine			
Business & Law	-0.232	0.091	**0.011**
Education & Arts	-0.043	0.076	0.571
Engineering & Built Environment	-0.048	0.100	0.634
Science	-0.045	0.086	0.599
Living situation			
Reference: Parents' home			
Own home	-0.024	0.115	0.836
On-campus residences	-0.053	0.109	0.626
Renting	-0.133	0.069	0.056
Others	-0.107	0.179	0.551
Receiving financial support	-0.001	0.060	0.991
Not meeting vegetable recommendations	-0.125	0.097	0.197
Age	0.010	0.005	**0.027**
Male	-0.209	0.067	**0.002**
International	-0.230	0.127	0.071
Faculty of study			
Reference: Health & Medicine			
Business & Law	-0.205	0.092	**0.025**
Education & Arts	-0.045	0.077	0.561
Engineering & Built Environment	-0.071	0.101	0.481
Science	-0.060	0.087	0.492
Living situation			
Reference: Parents' home			
Own home	0.031	0.115	0.786
On-campus residences	-0.054	0.110	0.623
Renting	-0.125	0.070	0.073

Others	-0.096	0.180	0.596
Receiving financial support	0.025	0.060	0.674
Soft drink > 1 cup per week	-0.307	0.066	**< 0.001**
Age	0.007	0.005	0.128
Male	-0.198	0.067	**0.003**
Non-ATSI	0.003	0.142	0.984
International	-0.328	0.132	**0.013**
Postgraduate	0.323	0.101	**0.001**
Faculty of study			
Reference: Health & Medicine			
Business & Law	-0.200	0.094	**0.033**
Education & Arts	Automatically excluded by SPSS		
Engineering & Built Environment	-0.039	0.102	0.704
Science	-0.015	0.088	0.863
Year of program			
Reference: 1st year			
2nd year	0.093	0.077	0.226
3rd year	-0.045	0.078	0.564
4th year	0.055	0.095	0.563
5th year and above	0.015	0.108	0.889
Living situation			
Reference: Parents' home			
Own home	0.102	0.106	0.337
On-campus residences	0.110	0.112	0.324
Renting	Automatically excluded by SPSS		
Others	0.074	0.179	0.681
Paid work (hours/week)	-0.001	0.002	0.684
Takeaway food ≥ 1 time(s) per week	-0.130	0.060	**0.029**
Age	0.007	0.005	0.148
Male	-0.213	0.067	**0.001**
Non-ATSI	0.002	0.142	0.989
International	-0.347	0.129	**0.007**
Postgraduate	0.320	0.096	**0.001**
Faculty of study			
Reference: Health & Medicine			
Business & Law	-0.222	0.092	**0.016**
Education & Arts	-0.037	0.077	0.629
Engineering & Built Environment	-0.060	0.101	0.555

Science	-0.044	0.087	0.611
Living situation			
Reference: Parents' home			
Own home	-0.014	0.116	0.902
On-campus residences	-0.054	0.107	0.615
Renting	-0.141	0.069	**0.042**
Others	-0.095	0.179	0.596
No daily breakfast consumption	-0.261	0.057	**< 0.001**
Age	0.008	0.005	0.108
Male	-0.215	0.066	**0.001**
International	-0.310	0.129	**0.017**
Postgraduate	0.297	0.098	**0.002**
Faculty of study			
Reference: Health & Medicine			
Business & Law	-0.200	0.092	**0.029**
Education & Arts	-0.008	0.077	0.916
Engineering & Built Environment	-0.041	0.100	0.683
Science	-0.022	0.086	0.801
Year of program			
Reference: 1st year			
2nd year	0.096	0.077	0.214
3rd year	-0.060	0.078	0.443
4th year	0.044	0.094	0.639
5th year & above	0.047	0.107	0.661
Living situation			
Reference: Parents' home			
Own home	-0.021	0.115	0.852
On-campus residences	-0.023	0.107	0.831
Renting	-0.129	0.069	0.062
Others	-0.074	0.179	0.679
Not meeting physical activity recommendations	-0.010	0.064	0.877
Age	0.008	0.005	0.097
Male	-0.230	0.067	**0.001**
International	-0.332	0.131	**0.011**
Postgraduate	0.319	0.099	**0.001**
Faculty of study			
Reference: Health & Medicine			
Business & Law	-0.231	0.093	**0.013**

Education & Arts	-0.046	0.077	0.550
Engineering & Built Environment	-0.068	0.101	0.501
Science	-0.045	0.087	0.608
Year of program			
Reference: 1st year			
2nd year	0.099	0.077	0.199
3rd year	-0.043	0.079	0.586
4th year	0.062	0.095	0.517
5th year & above	0.054	0.108	0.619
Living situation			
Reference: Parents' home			
Own home	-0.020	0.116	0.863
On-campus residences	-0.043	0.110	0.696
Renting	-0.144	0.070	**0.040**
Others	-0.098	0.180	0.585
Receiving financial support	0.011	0.060	0.853
Sitting time > 8 hours/day	-0.011	0.060	0.851
Age	0.011	0.005	**0.018**
Male	-0.228	0.067	**0.001**
Australian born	0.096	0.087	0.267
Faculty of study			
Reference: Health & Medicine			
Business & Law	-0.166	0.095	0.081
Education & Arts	Automatically excluded by SPSS		
Engineering & Built Environment	-0.018	0.103	0.863
Science	-0.002	0.089	0.980
Year of program			
Reference: 1st year			
2nd year	0.098	0.078	0.206
3rd year	-0.081	0.078	0.301
4th year	0.019	0.095	0.840
5th year & above	0.009	0.107	0.930
Living situation			
Reference: Parents' home			
Own home	0.024	0.117	0.839
On-campus residences	-0.082	0.108	0.450
Renting	-0.143	0.069	**0.040**
Others	-0.115	0.181	0.525

Paid work (hours/week)	0.002	0.002	0.529
Alcohol (Exceeding single occasion risk)	-0.277	0.062	**< 0.001**
Age	0.005	0.005	0.287
Male	-0.211	0.066	0.287
International	-0.395	0.132	**0.003**
Postgraduate	0.312	0.101	**0.002**
Faculty of study			
Reference: Health & Medicine			
Business & Law	-0.169	0.094	0.073
Education & Arts	Automatically excluded by SPSS		
Engineering & Built Environment	-0.024	0.101	0.811
Science	-0.004	0.088	0.968
Year of program			
Reference: 1st year			
2nd year	0.101	0.077	0.168
3rd year	-0.038	0.078	0.624
4th year	0.048	0.095	0.609
5th year and above	0.065	0.107	0.545
Living situation			
Reference: Parents' home			
Own home	0.124	0.106	0.242
On-campus residences	0.145	0.112	0.196
Renting	Automatically excluded by SPSS		
Others	0.057	0.179	0.749
Paid work (hours/week)	< 0.001	0.002	0.989
Smoker	-0.393	0.113	**0.001**
Age	0.011	0.005	**0.018**
Male	-0.243	0.062	**0.000**
Non-ATSI	-0.023	0.143	0.874
Year of program			
Reference: 1st year			
2nd year	0.107	0.077	0.167
3rd year	-0.091	0.078	0.242
4th year	0.024	0.094	0.802
5th year and above	-0.018	0.106	0.865
Living situation			
Reference: Parents' home			
Own home	0.036	0.115	0.752

On-campus residences	-0.077	0.105	0.463
Renting	-0.130	0.069	0.059
Others	-0.110	0.180	0.540
Not meeting sleep recommendations	-0.163	0.068	**0.018**
Age	0.013	0.005	**0.007**
Male	-0.246	0.062	**0.000**
Year of program			
Reference: 1st year			
2nd year	0.102	0.077	0.189
3rd year	-0.079	0.078	0.311
4th year	0.023	0.094	0.807
5th year and above	-0.023	0.107	0.828
Living situation			
Reference: Parents' home			
Own home	0.018	0.115	0.877
On-campus residences	-0.088	0.105	0.405
Renting	-0.154	0.068	**0.024**
Others	-0.130	0.180	0.469
Risk Factor Score	-0.105	0.026	**< 0.001**
Age	0.012	0.004	**0.001**
Male	-0.206	0.067	**0.002**
Non-ATSI	0.018	0.142	0.897
International	-0.268	0.124	**0.030**
Faculty of study			
Reference: Health & Medicine			
Business & Law	-0.172	0.092	0.061
Education & Arts	-0.014	0.076	0.855
Engineering & Built Environment	-0.030	0.101	0.768
Science	-0.023	0.087	0.792
Year of program			
Reference: 1st year			
2nd year	0.097	0.077	0.206
3rd year	-0.086	0.077	0.268
4th year	0.000	0.093	0.996
5th year and above	-0.032	0.106	0.763
Receiving financial support	0.031	0.058	0.592

ATSI; Aboriginal or Torres Strait Islander, [a] β-Coefficient indicates the increase in the dependent variable per unit increase in the independent variable. Significant p-values in **bold**.
